# Forms of Calling and Helping Behaviors at Work: Psychological Entitlement and Moral Duty as Mediators

**DOI:** 10.3390/bs14111029

**Published:** 2024-11-02

**Authors:** Sang Woo Park, Young Woo Sohn

**Affiliations:** Department of Psychology, Yonsei University, Seoul 03722, Republic of Korea; swpark1027@yonsei.ac.kr

**Keywords:** modern calling, neoclassical calling, psychological entitlement, moral duty, unethical pro-organizational behavior, organizational citizenship behavior

## Abstract

Even though calling researchers have identified two major forms of calling, namely modern and neoclassical, existing studies do not agree on whether these two forms are consistent or different in their mechanisms and outcomes. This study aimed to investigate whether modern and neoclassical calling are both indirectly related to unethical pro-organizational behavior (UPB) and organizational citizenship behavior (OCB) through the mediating roles of psychological entitlement and moral duty. Additionally, this study also aimed to investigate whether psychological entitlement functions as a mediator greater in magnitude for modern calling, whereas moral duty functions as a mediator greater in magnitude for neoclassical calling. Results from 463 employees in South Korea from two time points at 1 month interval revealed that both modern and neoclassical calling were indirectly positively related to UPB through psychological entitlement and indirectly positively related to OCB through moral duty. There was insufficient evidence to support the notion that the mediators would be different in magnitude based on the form of calling. Thus, this study demonstrates the double-edged nature of calling in relation to OCB and UPB. Additionally, it suggests that the outcomes of employees’ calling at work may depend more on the strength rather than the form of their calling. The implications and directions for future research are discussed.

## 1. Introduction

Do modern calling and neoclassical calling lead to consistent or distinct mechanisms and outcomes? In existing research, calling can be broadly categorized into modern calling and neoclassical calling, also known as internal calling and external calling, respectively. Although researchers have actively studied calling as an empirically definable construct over the past three decades, the answer to this question remains disputed [1,2,3]. Among these researchers, Dobrow et al. [3] suggested in their theory on calling and good life that while modern and neoclassical calling have shared mechanisms and outcomes, self-oriented mechanisms are more important for modern calling, whereas other-oriented mechanisms are more important for neoclassical calling.

In this study, based on Dobrow et al.’s [3] theory, psychological entitlement and moral duty are presented as contrasting mediators in terms of self- and other-orientations, with unethical pro-organizational behavior (UPB) and organizational citizenship behavior (OCB) presented as contrasting outcomes. Altogether, this study will investigate whether the two forms of calling indeed have shared mechanisms and outcomes and whether modern calling is characterized by a self-orientation pathway that is more important, while neoclassical calling is characterized by an other-orientation pathway that is more important. UPB and OCB were selected as the outcomes of the study, as they are variations of helping behaviors previously shown to be related to calling [4,5]. The use of two variations of helping behaviors instead of one was intended to improve the generalizability of the findings.

## 2. Literature Review and Hypotheses

### 2.1. Modern and Neoclassical Calling

In their seminal empirical work on calling, Wrzesniewski et al. [6] proposed a definition in which a calling refers to work that is primarily achieved for personal fulfillment and not for material gains or career advancements. Nevertheless, Wrzesniewski et al.’s definition of calling was different from the classical definition of calling that heavily emphasizes the presence of an external caller and the call of duty, and as such, not all researchers employed it [1,7,8]. This disagreement in defining contemporary calling eventually manifested itself as research on calling being separated into two major branches, with the form of calling defined in Wrzesniewski et al.’s manner being called modern calling and the other form of calling that is closer to the classical definition of calling being called neoclassical calling [3,7,9].

Following Wrzesniewski et al.’s [6] proposition, Dobrow and Tosti-Kharas [10] defined modern calling as “a consuming, meaningful passion people experience toward a domain” (p. 1001). In this sense, a calling is a subjective psychological phenomenon that deals with one’s internal drive toward a specific domain of work and is not ultimately defined by the request or demand of any outside source [10]. Modern calling has been positively associated with numerous positive work-related variables, including job satisfaction [6], self-efficacy [10], career commitment [9], and proactive professional development [11], and negatively associated with deleterious variables, such as the number of days missed at work [6] and turnover intention [12].

Dik and Shimizu [13] suggested that modern calling is a natural outgrowth of the shift of contemporary culture from a religious to a secular one, as well as the shift from one that values the common good to one that emphasizes the individual as its higher good. One common criticism of this formulation of calling is that by reducing calling to an expression of internal passion, modern calling does not directly take into account the relationship between the caller and the called [8]. In this line of argument, Bunderson and Thompson [7] suggested that a calling forged merely through one’s personal passion and enjoyment is easily breakable, whereas a calling forged by duty and purpose becomes truly binding and noble. It is this latter part that the other branch of calling, namely neoclassical calling, tries to address.

As can be seen from the prefix neo, neoclassical calling aims to inherit the classical understanding of calling, but unlike classical calling, which only includes religious sources as legitimate sources of one’s calling, neoclassical calling utilizes a broader definition that also includes the call of duty from non-religious sources (e.g., family, nation) as valid sources of calling.

Dik and Duffy’s [14] representative definition of neoclassical calling describes it as a type of work attitude that acknowledges the presence of an outside source that drew one to one’s work, with this work being carried out for other-oriented values and goals (e.g., being helpful to others). The key point that separates neoclassical calling from modern calling is the idea of transcendent summons. In other words, unlike modern calling, which focuses on personal inner fulfillment, neoclassical calling places heavier emphasis on the fulfillment of external requirements and needs [2].

Neoclassical calling has been positively associated with numerous positive work-related variables, including work hope [15], job satisfaction [16], and job performance [4], and negatively associated with deleterious variables such as burnout [16].

In terms of how modern and neoclassical calling may operate differently, Dobrow et al. [3] proposed a theoretical model that identifies potential mechanisms that may play differential roles for each form of calling. This theoretical model of the relationship between calling and good life identifies two primary pathways by which calling is related to its outcomes, namely self-realization and unification (see also Rosso et al. [17]). Self-realization refers to the fulfillment of self-oriented needs (e.g., autonomy, competence, and uniqueness), whereas unification refers to the fulfillment of other-oriented needs (e.g., relatedness and social worthiness). Through these two primary pathways, a calling is predicted to relate to proximal work outcomes (e.g., job satisfaction and work meaningfulness). Specifically, a modern calling’s primary pathway is expected to be based on self-orientation, whereas a neoclassical calling’s primary pathway is expected to be based on other-orientation. While both forms of calling are expected to function through secondary pathways (i.e., modern calling through other-orientation and neoclassical calling through self-orientation), the primary pathway for each form of calling is expected to be stronger than the respective secondary pathway [3].

Based on Dobrow et al.’s [3] theoretical framework, in this study, both modern and neoclassical calling are expected to positively relate to helping behaviors at work in the form of OCB and UPB, with psychological entitlement and moral duty functioning as mediators. Additionally, for modern calling, psychological entitlement is expected to be the mediator greater in magnitude, whereas for neoclassical calling, moral duty is expected to be the mediator greater in magnitude.

### 2.2. The Relationship Between Calling and OCB/UPB

#### 2.2.1. Calling and OCB

OCB refers to behaviors at work that are helpful to one’s organization and its members, but are not formally recognized or rewarded [18]. Examples of such behaviors include investing time to listen to the concerns of other employees or not taking unwarranted work breaks, which may be desirable for organizations seeking productive employees [19].

Since OCB is a construct that is directly related to altruism, courtesy, and interpersonal harmony, the prosocial aspect of neoclassical calling is expected to positively relate to OCB, such that those with higher neoclassical calling will manifest higher OCB. Indeed, across various occupations, such as sales workers [4] and bank employees [20], neoclassical calling has been shown to be positively related to OCB.

As for modern calling, it is possible that a deep sense of passion and enjoyment of one’s work will lead to work-related behaviors that also happen to be helpful to one’s organization and members [21]. Past work has supported this relationship between calling and OCB [3,22]. As such, in this study, both modern and neoclassical calling are expected to relate positively to OCB.

#### 2.2.2. Calling and UPB

UPB refers to unethical behaviors that are consciously conducted to assist one’s organization and members [23]. Examples of such behaviors include intentionally hiding damaging information about one’s organization or deceitfully exaggerating the quality of one’s organization’s products [24]. While OCB and UPB are similar in the sense that both refer to helping behaviors at work, UPB is distinct from OCB due to UPB’s explicit aspect of violating social norms and ethical standards [24]. In addition, unlike OCB, whether employers will find the UPB of their employees desirable is a questionable matter, since it may ultimately result in serious financial or legal hazards for the organization [24].

At first glance, a calling may seem unlikely to be positively related to UPB, given its predominant association with good-willed endeavors [2,3,7]. For example, in a previous study, modern calling and UPB were found to be negatively related [25]. As for neoclassical calling, while there has been no empirical study yet on the relationship between neoclassical calling and UPB, the other-oriented, prosocial aspect of neoclassical calling suggests that the relationship between the two would also be negative [26].

### 2.3. Psychological Entitlement and Moral Duty as Mediators

Nevertheless, the indirect relationship between the two forms of calling and UPB may ultimately depend on the mediating mechanisms. In this study, the hypothesized set of mechanisms are psychological entitlement and moral duty.

Psychological entitlement and moral duty are a set of mechanisms that have been put into contrast with one another in various forms under different names, such as entitlement versus obligation [27], control versus self-abnegation [17], and rights versus responsibility [28]. In this sense, the placement of these two variables as a set of contrasting mediators is a decision supported by past research. In this study, psychological entitlement represents a form of self-orientation, while moral duty represents a form of other-orientation.

#### 2.3.1. The Mediating Role of Psychological Entitlement

Psychological entitlement refers to a heightened view of the self in which individuals believe they deserve highly favorable treatment and outcomes [29]. While the construct is sometimes studied as a trait that people have [30], it is also frequently studied as a state-like attitude that can change in reaction to life and work experiences [31,32]. Psychological entitlement is not a construct that is by necessity harmful, as it could be a fair reflection of the need for one to be treated as a human being with rights and dignity. Nevertheless, the literature on psychological entitlement often indicates that the construct is linked to negative attributes and outcomes such as work frustration [33] and lower job satisfaction [34].

In this study, modern calling is expected to be positively related to psychological entitlement. To date, no study has directly examined the relationship between the two. Nevertheless, past work on the relationship between calling-related constructs and psychological entitlement suggests that modern calling may indeed be positively related to psychological entitlement. For example, Lafrenière et al. [35] observed that obsessive forms of passion are positively related to psychological entitlement. Past research has also demonstrated that modern calling may be ego-driven and related to the desire for self-enhancement [36,37,38]. Based on these suggestions, in this study, modern calling is expected to be positively related to psychological entitlement.

Regarding the relationship between neoclassical calling and psychological entitlement, previous research suggests that fulfilling the demands of an external source through one’s work may be viewed as a justification for why one should receive favorable treatment and outcomes [31,39]. Indeed, it is perhaps natural for one to expect to be rewarded with good outcomes for doing good [39]. As such, in this study, neoclassical calling is expected to be positively related to psychological entitlement.

Concerning psychological entitlement’s direct relationship with UPB, past research has demonstrated that psychological entitlement is related to a greater propensity to engage in UPB [40,41,42]. A. Lee et al. [41] suggested that this is due to the combination of moral rationalization and ego-defense that high psychological entitlement entails, in which psychologically entitled employees view UPB as a form of shortcut by which they can demonstrate their deservingness of favorable treatments by their willingness to sacrifice greatly to advance the organization’s interests. As such, in this study, psychological entitlement is expected to be positively related to UPB.

As for psychological entitlement’s relationship with OCB, since psychological entitlement is a manifestation of a self-serving inclination, it is likely that such an inclination would be directly related to fewer acts of ethically helpful behavior toward one’s organization [40]. As such, in this study, psychological entitlement is expected to be negatively related to OCB.

To summarize, both modern and neoclassical calling are expected to relate positively to psychological entitlement. In turn, psychological entitlement is expected to relate positively to UPB but negatively related to OCB. This is in line with the theoretical relationship suggested by Dobrow et al. [3], in which both forms of calling are related to work outcomes (i.e., UPB, OCB) mediated by a mechanism characterized by self-orientation (i.e., psychological entitlement). Based on this discussion, we propose the following hypotheses:

**Hypothesis** **1a.** *The positive effect of modern calling on UPB is mediated by psychological entitlement.*

**Hypothesis** **2a.** *The negative effect of modern calling on OCB is mediated by psychological entitlement.*

**Hypothesis** **3a.** *The positive effect of neoclassical calling on UPB is mediated by psychological entitlement.*

**Hypothesis** **4a.** *The negative effect of neoclassical calling on OCB is mediated by psychological entitlement.*

#### 2.3.2. The Mediating Role of Moral Duty

Moral duty refers to the belief that one has been given responsibility to work ethically [7,27]. In contrast to psychological entitlement, moral duty is the mediator representing other-orientation in this study. The concept itself has been associated with numerous positive outcomes such as greater work engagement [43] and collaboration with others [44].

In this study, modern calling is expected to be positively related to moral duty. It is likely that a consuming passion for one’s work will motivate one to take care of it by working in a way that ensures its longevity and does not jeopardize it. Indeed, Michaelson and Tosti-Kharas [45] reported that the participants in their study viewed self-oriented callings as moral and ethical.

As for neoclassical calling, it is expected to positively relate to moral duty as well. The idea that an external summons naturally leads one to experience a sense of obligation and ethical duty has been a common component in various definitions of neoclassical calling, and has been empirically supported in past research [2,7]. In line with this past work, neoclassical calling is expected to relate positively to moral duty in this study.

Regarding moral duty’s direct relationship with UPB, the two are expected to be negatively related in this study. Indeed, in past research, variables related to moral duty, such as felt obligation and moral identity, were negatively related to unethical behaviors at work [42,46]. As such, in this study, moral duty is expected to be negatively related to UPB.

Concerning moral duty’s relationship with OCB, moral duty is expected to relate positively to it. Since moral duty refers to experiencing a sense of necessity to not only work but also work in an ethical manner, those who experience a high degree of moral duty will be naturally inclined to engage in prosocial behaviors that benefit their organization and members. This relationship was supported in past research [27,44]. As such, in this study, moral duty is expected to be positively related to OCB.

To summarize, both modern and neoclassical calling are expected to relate positively to moral duty. In turn, moral duty is expected to be negatively related to UPB but positively related to OCB. This is in line with the theoretical relationship suggested by Dobrow et al. [3], in which both forms of calling are related to work outcomes (i.e., UPB, OCB) mediated by a mechanism characterized by other-orientation (i.e., moral duty). Based on this discussion, we propose the following hypotheses:

**Hypothesis** **1b.** *The negative effect of modern calling on UPB is mediated by moral duty.*

**Hypothesis** **2b.** *The positive effect of modern calling on OCB is mediated by moral duty.*

**Hypothesis** **3b.** *The negative effect of neoclassical calling on UPB is mediated by moral duty.*

**Hypothesis** **4b.** *The positive effect of neoclassical calling on OCB is mediated by moral duty.*

### 2.4. Potential Differences in Mechanisms for Modern and Neoclassical Calling

**Figure 1:**
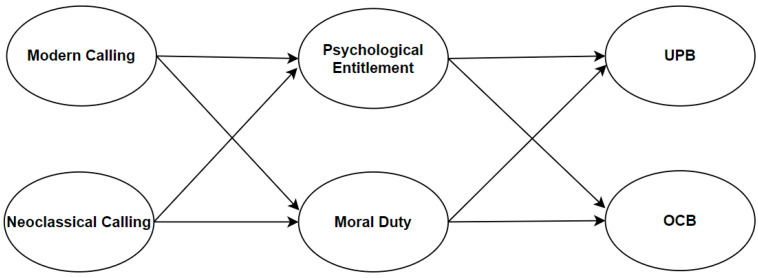
Hypothesized model.

According to Dobrow et al.’s [3] theory, while both forms of calling are related to work-relevant outcomes through mechanisms of self- and other-orientations, for modern calling, self-orientation is expected to be the stronger mechanism compared to other-orientation, whereas for neoclassical calling, other-orientation is expected to be the stronger mechanism compared to self-orientation. Applied to this study, this would mean that modern calling’s indirect effect on the two outcomes (UPB and OCB) will be greater in magnitude when psychological entitlement is the mediator compared to moral duty, whereas neoclassical calling’s indirect effect on the two outcomes will be greater in magnitude when moral duty is the mediator compared to psychological entitlement. In accordance with Dobrow et al.’s framework, this potential distinction in mediators will also be tested in this study. Based on this discussion, we propose the following hypotheses (see Figure 1 for the hypothesized model):

**Hypothesis** **1c.** *The indirect effect of modern calling on UPB is greater in magnitude when mediated by psychological entitlement than by moral duty.*

**Hypothesis** **2c.** *The indirect effect of modern calling on OCB is greater in magnitude when mediated by psychological entitlement than by moral duty.*

**Hypothesis** **3c.** *The indirect effect of neoclassical calling on UPB is greater in magnitude when mediated by moral duty than by psychological entitlement.*

**Hypothesis** **4c.** *The indirect effect of neoclassical calling on OCB is greater in magnitude when mediated by moral duty than by psychological entitlement.*

## 3. Materials and Methods

### 3.1. Participants and Procedure

This study was approved by the Institutional Review Board (IRB) of the authors before data collection. The participants for the study were recruited via an online recruitment platform called dataSpring. The study was conducted in a time-lagged manner consisting of two waves (i.e., T1 and T2), with each wave being 1 month apart. The participants provided their responses by completing an online survey hosted on the recruitment platform’s system. After the first survey (T1) was administered, the second survey (T2) was conducted one month later. To improve the sampling procedure, aside from the requirement that participants be employed adults, no other pre-determined limits (e.g., occupation, tenure) were set.

The first wave consisted of responses from 500 adult South Korean employees. Among these, 37 responses were removed from subsequent analyses due to failing the attention check test. The second wave consisted of 342 responses with a 73.87% response rate. The mean age of the participants was 43.79 (*SD* = 12.84). 49.24% were male and 50.76% were female. The mean tenure of the participants was 106.71 months (*SD* = 100.58).

### 3.2. Measures

#### 3.2.1. Modern Calling

Modern calling was measured using the following item developed for this study: “I have a very strong passion for my job, and I think about my job so often that I do so even when I am not at work. Additionally, I enjoy my work much more than most other things in life”. The content of the item was based on past discussions and measures of modern calling in the literature, as well as feedback from experts in the field [2,3,6,10,47]. Past studies have also measured calling with single-item paragraph measures, and the use of such measures is an established practice in the calling literature [6,48,49]. This study used an exploratory set of single-item paragraph measures for modern and neoclassical calling, since previous measures were not designed to capture the distinctions between the two forms [3,6,48,49]. Responses were recorded on a 7-point Likert-type scale ranging from 1 (strongly disagree) to 7 (strongly agree). A higher score indicates a higher degree of modern calling.

#### 3.2.2. Neoclassical Calling

Neoclassical calling was measured using the following item developed for this study: “I have been drawn to my job by an external source, with this external source being religious reasons, familial duty, or national and societal needs. I need to do my work to fulfill the expectations of this external source”. The content of the item was based on past discussions and measures of neoclassical calling in the literature [2,3,7,15,47]. Responses were given on a 7-point Likert-type scale ranging from 1 (strongly disagree) to 7 (strongly agree). A higher score indicates a higher degree of neoclassical calling.

#### 3.2.3. Psychological Entitlement

Psychological entitlement was measured with six items from E. R. Lee’s [50] Korean translation of Campbell et al.’s [29] Psychological Entitlement Scale (PES). Responses were recorded on a 5-point Likert-type scale ranging from 1 (strongly disagree) to 5 (strongly agree). An example item is, “I feel entitled to more of everything”. In E. R. Lee’s study, Cronbach’s alpha (α) was 0.86. In this study, McDonald’s omega (ω) was 0.84. A higher total score indicates a higher degree of psychological entitlement.

#### 3.2.4. Moral Duty

Moral duty was measured using four items based on Ham et al.’s [51] Korean translation of the moral duty measure developed by Bunderson and Thompson [7]. Responses were given on a 5-point Likert-type scale ranging from 1 (strongly disagree) to 5 (strongly agree). The wordings of the items were adjusted to make the moral aspect of the items more explicit. An example item is, “I consider it my sacred duty to do all I ethically can for my work”. In Ham et al.’s study, Cronbach’s alpha (α) was 0.85. In this study, McDonald’s omega (ω) was 0.85. A higher total score indicates a higher degree of moral duty.

#### 3.2.5. UPB

UPB was measured using four items from K. Lee and Jeon’s [52] Korean translation of the UPB measure developed by Umphress et al. [53]. Responses were given on a 5-point Likert-type scale ranging from 1 (strongly disagree) to 5 (strongly agree). An example item is, “If it would help my organization, I would exaggerate the truth about my company’s products or services to customers and clients”. In K. Lee and Jeon’s study, Cronbach’s alpha (α) was 0.84. In this study, McDonald’s omega (ω) was 0.86 at T1 and 0.84 at T2. A higher total score indicates a higher degree of UPB.

#### 3.2.6. OCB

OCB was measured with eight items from Kim’s [54] Korean translation of the OCB measure developed by Williams and Anderson [18]. Responses were given on a 5-point Likert-type scale ranging from 1 (strongly disagree) to 5 (strongly agree). An example item is, “Helps others who have heavy workloads”. In Kim’s [54] study, Cronbach’s alpha (α) was 0.83. In this study, McDonald’s omega (ω) was 0.84 at T1 and 0.84 at T2 as well. A higher total score indicates a higher degree of OCB.

### 3.3. Statistical Analyses

The hypotheses of the study were examined using structural equation modeling with the lavaan package [55] in R version 4.4.1 [56]. Missing data were handled with full information maximum likelihood (FIML). In line with past studies on calling, demographic variables (age, sex, and tenure) were entered as control variables [4].

## 4. Results

### 4.1. Preliminary Analyses

#### 4.1.1. Attrition Analysis

Multiple logistic regression was used to check whether the responses from participants who answered at both T1 and T2 were different from those who answered only at T1 [57]. Results showed that there were no statistically significant differences between the groups for the study’s variables, including demographics.

#### 4.1.2. Measurement Invariance over Time

To check if UPB and OCB were measured similarly at T1 and T2, measurement invariance was tested by comparing the configural model to the metric model, the metric model to the scalar model, and the scalar model to the residual model. Changes in model fit were considered to be practically significant for the following criteria: ∆CFI = −0.010, ∆RMSEA = 0.015, and ∆SRMR = 0.010 [58]. The changes in model fit were all below the pre-determined threshold, thereby demonstrating measurement invariance over time.

#### 4.1.3. Descriptive Statistics and Correlations

The descriptive statistics and correlations of the variables are shown in Table 1. The skewness and kurtosis of the variables were all lower than the pre-determined thresholds of |3| and |10|, respectively, suggesting that non-normality was not an issue [59]. Modern calling was positively correlated with all the main study variables, including neoclassical calling (*r* = 0.41), psychological entitlement (*r* = 0.30), moral duty (*r* = 0.35), UPB T2 (*r* = 0.11), and OCB T2 (*r* = 0.34). Neoclassical calling was also positively correlated with all main study variables, including psychological entitlement (*r* = 0.27), moral duty (*r* = 0.28), UPB T2 (*r* = 0.14), and OCB T2 (*r* = 0.32).

### 4.2. Measurement Model

Confirmatory factor analysis was performed to test a six-factor measurement model that included psychological entitlement, moral duty, OCB T1, OCB T2, UPB T1, and UPB T2 (modern and neoclassical calling were not included since they were not measured as latent variables). The error terms of identical items from different time points were set to correlate with one another. The fit of the model was considered acceptable if it met the following criteria: CFI ≥ 0.90, RMSEA ≤ 0.10, and SRMR ≤ 0.10, whereas the fit of the model was considered good if it met the following criteria: CFI ≥ 0.95, RMSEA ≤ 0.06, and SRMR ≤ 0.08 [59,60]. Results showed that the model fit of the six-factor model was acceptable: χ^2^ (500) = 824.457, CFI = 0.930, RMSEA = 0.044, SRMR = 0.050.

An alternative five-factor model in which psychological entitlement and moral duty were combined into one factor and a second alternative five-factor model in which UPB T2 and OCB T2 were combined into one factor were compared against the original six-factor model. Changes in model fit were considered practically significant for the following criteria: ∆CFI = −0.010, ΔRMSEA = 0.015, and ΔSRMR = 0.010 [58]. The first alternative model (∆CFI = −0.119, ∆RMSEA = 0.027, ∆SRMR = 0.044) and the second alternative model (∆CFI = −0.176, ∆RMSEA = 0.037, ∆SRMR = 0.048) both exhibited a worse fit when compared to the original model.

### 4.3. Structural Model

**Figure 2:**
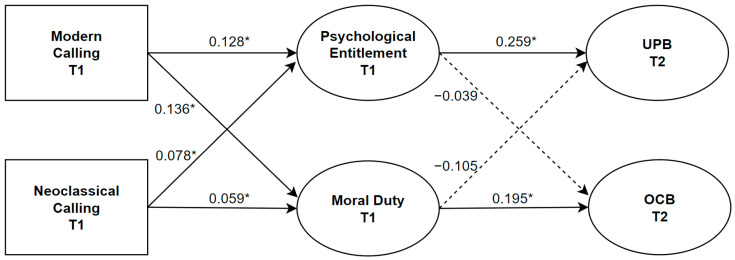
Structural model. *Note.* The control variables (age, sex, tenure), autoregressive paths, and direct paths from calling (modern, neoclassical) to UPB and OCB are not included in the figure for clarity. Estimates are in unstandardized beta (*b*). Dashed lines indicate non-significant paths. * = *p* < 0.05. Squares indicate observed variables. Circles indicate latent variables.

In the structural model based on the initial six-factor measurement model, modern and neoclassical calling were set to have paths toward the two mediators (psychological entitlement and moral duty), as well as the two outcomes (UPB and OCB). In turn, the two mediators were set to have paths toward the two outcomes. The three demographic control variables (age, sex, and tenure) were also set to have paths toward both mediators and outcomes. Autoregressive paths for UPB and OCB were also included. This model showed an acceptable fit to the data: χ^2^ (647) = 1126.940, CFI = 0.922, RMSEA = 0.040, and SRMR = 0.066 (see Figure 2 for the structural model).

### 4.4. Hypotheses Testing

To test the hypothesized indirect effects, bootstrapped samples (10,000) were generated using the percentile method and were considered statistically significant if their 95% confidence intervals did not include zero [61]. The same criterion was used for the comparison of the indirect effects [62]. The indirect effects are shown in Table 2.

Hypotheses 1a, 2b, 3a, and 4b were supported because they did not include zero in their respective confidence intervals. By contrast, Hypotheses 1b, 2a, 3b, and 4a were not supported because they included zero in their respective confidence intervals. Finally, for indirect effect comparisons, Hypotheses 1c, 2c, 3c, and 4c were not supported because they included zero in their respective confidence intervals.

## 5. Discussion

This study examined whether modern and neoclassical calling are both related to UPB and OCB through psychological entitlement and moral duty. Additionally, this study sought to determine whether the magnitude of mediators would differ based on the form of calling. 

As hypothesized, both modern and neoclassical callings were positively related to psychological entitlement and moral duty. In turn, psychological entitlement was positively related to UPB, whereas moral duty was positively related to OCB. In other words, psychological entitlement mediated the positive relationship between modern/neoclassical calling and UPB, whereas moral duty mediated the positive relationship between modern/neoclassical calling and OCB. These results are consistent with past studies that have suggested a potential positive relationship between the two forms of calling and UPB when mediated by psychological entitlement [31,35,38,39]. These results are also consistent with past studies that have suggested a potential positive relationship between the two forms of calling and OCB when mediated by moral duty [7,27,44,45]. Together, these results contribute to the literature on calling by demonstrating that through the mediators, both forms of calling were not only related to a beneficial outcome in the form of OCB, but also related to a detrimental outcome in the form of UPB [7,11]. While previous studies have only shown this double-edged nature of calling in isolation, examining one form at a time [7,11,63], this study has further contributed by demonstrating this phenomenon with both forms of calling being simultaneously present.

Contrary to what was expected, psychological entitlement was not related to OCB. This contrasts with past studies that have suggested a negative relationship between the two [40]. However, it is important to note that there were also past studies in which the two were, on average, not related to one another [46,64]. As such, while not in line with what was initially expected in this study, this result supports the latter batch of studies in that experiencing greater psychological entitlement may not by itself be related to more future OCB.

Additionally, contrary to what was expected, moral duty was not related to UPB. This contrasts with past studies that have suggested a potential negative relationship between the two [42,46]. The current result suggests that an employee’s sense of moral duty may not by itself predict whether the employee will engage in either more or fewer UPB.

As for the comparison between psychological entitlement (self-orientation) and moral duty (other-orientation) as mechanisms for modern and neoclassical calling, the current results do not support Dobrow et al.’s [3] suggestion that self-orientation will be the mechanism that is greater in magnitude for modern calling, whereas other-orientation will be the mechanism that is greater in magnitude for neoclassical calling. More specifically, in all four hypothesized indirect relation comparisons for both modern and neoclassical calling, there were no statistically significant differences in the magnitudes of the mediators. In a previous study, Duffy et al. [48] approached the topic of calling in a way different from the current study, in which they analyzed calling as something that can come from three different sources: external summons, destiny, and fit. In their study, Duffy et al. observed that the three groups exhibited no differences in job satisfaction and life satisfaction. Other studies have also shared this sentiment that while modern and neoclassical calling do have genuine conceptual distinctions, they may not be clearly distinguished in what they predict [1,9,13]. The current results support this contrasting line of suggestion in the calling literature that even though the two forms of calling may have differences from a conceptual standpoint, these differences may not be extended to their mechanisms and outcomes. As Duffy et al. [1] once noted, “so far, results across research studies investigating correlates of calling have been extremely consistent regardless of how calling is conceptualized” (p. 425). This study has been added to the lineup of studies with very consistent results.

### 5.1. Practical Implications

The results of this study suggest that the benefits of having employees with a high sense of calling in terms of OCB may be consistent regardless of the calling’s form. As such, employers may not need to be concerned with the form of an employee’s calling, but rather its magnitude. Additionally, since past studies have shown that social support and clarity of professional identity are conducive to the development of a calling [65,66], it would be helpful for organizations to encourage and facilitate these elements in the workplace.

Nevertheless, since this study also demonstrated a dark side of calling, in which both forms of calling were related to UPB through psychological entitlement, organizations will also have to be mindful of this potential side effect that may reduce some of the benefits of calling. Previous work has suggested that psychological entitlement may increase in response to problematic work experiences such as relational conflict [67], perceived overqualification [68], and negative affect [69]. Additionally, past work has also suggested that the negative effects of psychological entitlement on employee behavior and performance may be mitigated by higher levels of ethical leadership [70] and trust at work [71]. As such, to minimize the potential side effects of calling in relation to psychological entitlement, employers should also strive to create a work environment that is characterized by an ethical work culture, a high sense of trust among employees, and ample opportunities for career development.

### 5.2. Limitations and Future Directions

There are several limitations to this study. First, while the two new measures used in this study for modern calling and neoclassical calling were developed after consulting the existing literature and measures of calling, they are nevertheless exploratory in the sense that more data are needed to support the assertion that these are valid measures that capture the distinctiveness of both forms of calling. Future studies should test the relationships between these measures and other calling-related variables to further demonstrate their validity.

Second, while the results of this study provide preliminary evidence that modern and neoclassical calling may not be differentially related to self- and other-orientations, there are many more sets of mechanisms (e.g., uniqueness vs. belongingness and authenticity vs. reputation) that can be applied to Dobrow et al.’s [3] framework. Future studies can incorporate different sets of mechanisms that were not used in this study to determine whether the pattern of results shown in this study holds.

Third, while this study utilized a time-lagged design consisting of measurements across two time points, it would have been more optimal to have three time points instead of two to best capture the processes implied in the theoretical model. While this study only utilized two time points due to constraints in resources, future studies could improve upon the design of this study by having measurements across more time points.

Fourth, this study recruited a sample consisting of employees in South Korea. While results concerning calling have been generally consistent across cultures [1], conducting future studies within the current framework in other cultures (e.g., Western cultures) will help improve the generalizability of the findings of this study.

## 6. Conclusions

This study demonstrates that psychological entitlement mediates the indirect effects of both modern and neoclassical calling on UPB and that moral duty mediates the indirect effects of both modern and neoclassical calling on OCB. Additionally, no difference was found between the magnitudes of each mediator for both modern and neoclassical callings. Thus, this study demonstrates the double-edged nature of both forms of calling and provides evidence for the notion that the outcomes of calling may be consistent, regardless of how they have been conceptualized.

## Figures and Tables

**Table 1:** Descriptive statistics and correlations.

Variable	*M*	*SD*	1	2	3	4	5	6	7	8	9	10
1. MC T1	4.25	1.32										
2. NC T1	3.97	1.62	0.41 **									
3. PE T1	3.29	0.66	0.30 **	0.27 **								
4. MD T1	3.71	0.67	0.35 **	0.28 **	0.24 **							
5. UPB T1	3.05	0.80	0.26 **	0.25 **	0.22 **	0.09						
6. UPB T2	3.09	0.77	0.11 *	0.14 **	0.28 **	0.06	0.58 **					
7. OCB T1	3.63	0.55	0.38 **	0.30 **	0.29 **	0.47 **	0.26 **	0.19 **				
8. OCB T2	3.64	0.54	0.34 **	0.32 **	0.19 **	0.48 **	0.31 **	0.24 **	0.66 **			
9. Age	43.79	12.84	0.22 **	0.17 **	−0.03	0.16 **	0.13 **	0.11 *	0.13 **	0.22 **		
10. Gender	0.51	0.50	−0.20 *	−0.16 **	−0.07	−0.08	−0.14 **	−0.07	−0.09 *	−0.13 *	−0.36 **	
11. Tenure	106.71	100.58	0.14 **	0.14 **	0.03	0.10 *	0.15 **	0.16 **	0.16 **	0.22 **	0.47 **	−0.31 **

*Note*. *N* = 463 at T1, 342 at T2. MC = modern calling. NC = neoclassical calling. PE = psychological entitlement. MD = moral duty. UPB = unethical pro-organizational behavior. OCB = organizational citizenship behavior. Sex: male = 0, female = 1. Tenure is in months. * *p* < 0.05. ** *p* < 0.01.

**Table 2:** Indirect effects from the structural model.

Indirect Effect (Hypothesis)	*b*	*SE*	95% CI
**1. Modern → Entitlement → UPB (H1a)**	**0.033**	**0.014**	**[0.010, 0.064]**
2. Modern → Duty → UPB (H1b)	−0.014	0.012	[−0.038, 0.009]
3. Modern → Entitlement → OCB (H2a)	−0.005	0.008	[−0.021, 0.009]
**4. Modern → Duty → OCB (H2b)**	**0.027**	**0.010**	**[0.009, 0.049]**
**5. Neoclassical → Entitlement → UPB (H3a)**	**0.020**	**0.009**	**[0.006, 0.039]**
6. Neoclassical → Duty → UPB (H3b)	−0.006	0.006	[−0.020, 0.004]
7. Neoclassical → Entitlement →OCB (H4a)	−0.003	0.005	[−0.013, 0.006]
**8. Neoclassical → Duty → OCB (H4b)**	**0.011**	**0.006**	**[0.002, 0.025]**
1 compared with 2 (H1c)	0.019	0.016	[−0.012, 0.050]
3 compared with 4 (H2c)	−0.021	0.012	[−0.044, 0.004]
5 compared with 6 (H3c)	0.014	0.010	[−0.021, 0.006]
7 compared with 8 (H4c)	−0.008	0.007	[−0.004, 0.034]

*Note.* 10,000 bootstrapped samples. Statistically significant relationships are indicated in bold. Differences in the absolute values of each indirect effect are reported for indirect effect comparisons.

## Data Availability

The data presented in the study are openly available at https://osf.io/9vjrx/, accessed on 1 November 2024.
